# Therapeutic effect of Duhuo Jisheng Decoction add-on Tui-na manipulation on osteoarthritis of knee: a randomized controlled trial

**DOI:** 10.1186/s13020-023-00737-5

**Published:** 2023-07-10

**Authors:** Kin Ho Chan, Jessica Y. L. Ching, Kam Leung Chan, Hoi Yi Lau, Ka Man Chu, Kenny Chan, Hon Fai Pang, Lok Chi Wong, Chon Pin Chia, Hong Wei Zhang, Tianhe Song, Sin Bond Leung, Bacon Fung Leung Ng, Zhi-Xiu Lin

**Affiliations:** 1 Yan Oi Tong-The Chinese University of Hong Kong Chinese Medicine Clinic Cum Training and Research Centre (Tuen Mun District), Hong Kong SAR, China; 2 Department of Medicine and Therapeutics, Faculty of Medicine, The Chinese University of Hong Kong, Shatin, N.T., Hong Kong SAR, China; 3 Hong Kong Institute of Integrative Medicine, Faculty of Medicine, The Chinese University of Hong Kong, Shatin, N.T., Hong Kong SAR, China; 4 School of Chinese Medicine, Faculty of Medicine, The Chinese University of Hong Kong, Shatin, N.T., Hong Kong SAR, China; 5 Yan Oi Tong-The University of Hong Kong Chinese Medicine Clinic Cum Training and Research Centre (Islands District), Hong Kong SAR, China; 6 Chinese Medicine Department, Hospital Authority, Hong Kong SAR, China

**Keywords:** Knee osteoarthritis, Knee pain, Tui-na, Chinese herbal medicine, Randomized controlled trial

## Abstract

**Background:**

Knee osteoarthritis (KOA) is a common degenerative joint condition that causes disability and pain in the elderly population. The prevalence of KOA among persons aged 63 or above is approximately 30%. Previous studies have reported the positive effects of Tui-na treatment and the Chinese herbal formula Du-Huo-Ji-Sheng Decoction (DHJSD) for KOA treatment. The current study aims to evaluate the add-on therapeutic effect of oral administration of DHJSD on KOA in addition to Tui-na.

**Methods:**

We conducted a prospective, randomized, controlled clinical trial. Seventy study subjects with KOA were randomly assigned to the treatment and control groups in a 1:1 ratio. Both two groups received eight sessions of Tui-na manipulation for 4 weeks. The DHJSD was only administered to the study subjects in the treatment group. The primary outcome measure was rated using the WOMAC at the end of treatment (4 weeks). Secondary outcomes were assessed using EQ-5D-5L, a health-related quality of life with 5-level EQ-5D version at end of treatment (week 4) and follow-up (week 8).

**Results:**

No statistically significant difference was found between two groups on WOMAC scores at the end of treatment. The mean WOMAC Pain subscale score was significantly lower in the treatment group than control group at week 8 follow up (mean difference, MD − 1.8, 95% CI − 3.5 to − 0.02, P = 0.048). The mean WOMAC Stiffness subscale score was significantly lower in the treatment group than in the control group at week 2 (MD 0.74, 95% CI 0.05 to 1.42, P = 0.035) and week 8 follow up (MD 0.95, 95% CI 0.26 to 1.65, P = 0.008). The mean EQ-5D index value was significantly improved in the treatment group than in the control group at week 2 (MD 0.17, 95% CI 0.02 to 0.31, P = 0.022). The analysis of WOMAC scores and EQ-5D-5L in both groups showed statistically significant improvement with time. No significant adverse effect was found during the trial.

**Conclusion:**

DHJSD may have an add-on effect in addition to Tui-na manipulation relieving pain and improving stiffness as well as quality of life (QOL) in patients with KOA. The combined treatment was generally safe and well tolerated.

*Trial registration* The study was registered at the ClinicalTrials.gov (website: https://clinicaltrials.gov/ct2/show/NCT04492670, registry number: NCT04492670), registered on 30 July 2020.

**Supplementary Information:**

The online version contains supplementary material available at 10.1186/s13020-023-00737-5.

## Background

Knee osteoarthritis (KOA) is a common degenerative joint condition and one of the most common causes of disability and pain amongst the elderly. The global age-standardized prevalence of knee OA was 3.8%, and the prevalence increases with age [1]. According to a recent study conducted in Hong Kong, by the year 2036, it is predicted that 30% of Hong Kong residents will be aged 65 or above [2], implying an estimated 10% of Hong Kong residents may suffer from KOA. Since the morbidity rate of KOA is extremely high in Hong Kong, it is of utmost important for us to establish an effective method to treat KOA.

The effectiveness of conventional therapies for KOA is limited for many reasons including an array of adverse side effects and drug resistance from the use of approved pharmacological drugs [3, 4]. Currently, the ultimate solution is surgery but prolonged waiting periods and effectiveness of total knee replacement (TKR) makes this option unfavorable. According to the data in 2021, the 90th percentile waiting time for total joint replacement surgery is approximately 62 months in Hong Kong [5]. Although mortality after primary TKR was low in public hospitals in Hong Kong [6], the probability of the occurrence of the postsurgical pain of TKR is approximately 53% [7].

Historically, traditional Chinese medicine techniques such as Tui-na were used to treat KOA and over the years this method has been refined and standardized. Clinical research has shown that Tui-na can relieve the pain, negative emotions, and disability of patients with KOA [8]. It has fewer associated adverse effects than other treatment schemes and is generally well accepted by patients.

Hong Kong is a “East meets West” place in which the Chinese medicine is very popular. Tui-na for KOA is highly demanded but owing to the limited number of Tui-na practitioners, the treatment cannot be fully maximized. There are 18 Tripartite Chinese Medicine Clinic cum Training and Research Centre supported by the Hospital Authority, 13 of them provide Tui-na services, but each Tui-na practitioner is only able to deliver the service to approximately 10 patients per day. With a view of maximizing the treatment effects so that the number of service sessions can be reduced, we aimed to explore a complementary treatment, Chinese herbal medicine, in additional to Tui-na to increase the synergistic effect. It was supposed to enhance the treatment effectiveness of Tui-na for patients with KOA.

Du-Huo-Ji-Sheng Decoction (DHJSD) is a widely used traditional Chinese herbal medicine for the treatment of arthritis in Asia [9, 10]. In animal studies, DHJSD has demonstrated significant anti-inflammatory effects through promoting lymphatic drainage function [11], and it can improve clinical symptoms, knee function and quality of life (QOL) for patients with KOA by inhibiting cartilage apoptosis [12]. In addition, DHJSD showed a lower risk of adverse events than standard western treatments [13]. There are very limited published reports about the clinical effectiveness of DHJSD. Hence this study aimed to evaluate the add-on clinical effectiveness of DHJSD in KOA. During the study, Tui-na was administered to the study subjects in both groups as a supportive treatment.

## Methodology

### Study design

This was a multicenter, prospective, randomized, controlled trial in patients with KOA. The study subjects were randomized to receive either Tui-na and DHJSD or Tui-na alone for 4 weeks, and followed-up 4 weeks after the end of treatment.

### Study population

Study subjects were recruited from the general public via daily outpatient services (Yan Oi Tong-The Chinese University of Hong Kong Chinese Medicine Clinic cum Training and Research Centre (Tuen Mun District) and Yan Oi Tong-The University of Hong Kong Chinese Medicine Clinic cum Training and Research Centre (Islands District) and advertisements in posters and leaflets from September 2020 to September 2021. The patients who were interested in participating in the study were referred to or self-approached the study sites to undergo eligibility assessment. They were pre-screened through a telephone interview. Potential study subjects for recruitment to the study had a face-to-face interview arranged to confirm the eligibility. During the interview, assessors explained the overall objectives and nature of the study, described the informed consent, and assessed the study subjects’ eligibility.

If the study subject had bilateral KOA and both knees fulfil the eligibility criteria, only the most symptomatic knee was treated and evaluated for the outcome assessment throughout the whole study period. The relatively mild side of knee was treated after the end of study as compensation.

### Eligibility criteria

Study subjects aged 50 years or older, met the criteria of KOA according to the American College of Rheumatology [14], with Western Ontario and McMaster University Osteoarthritis Index (WOMAC) [15] score 39 or above were recruited with a willingness to participate in the study.

We excluded study subjects who had other disorders that might affect the knee (e.g., infection, malignant or autoimmune diseases); who had knee surgery or arthroscopy in the past year; who had knee chondroprotective or intra-articular injection, or received systemic corticoid treatment in the past 4 months; who had local antiphlogistic treatment, acupuncture, or physiotherapy in the past 2 weeks; who took anticoagulants, antiplatelets, corticosteroids, psychiatric drugs, hormones, antiarrhythmic drugs or diuretics drug; who had uncontrolled hypertension; who had comorbidities including severe cardiovascular, cerebral, hepatic, renal, or hematopoietic diseases; who had a history of mental illness; or who had allergic reaction to or had drug interaction with the study herbs.

### Randomization and masking

Before randomization, there were 2 weeks screening period for pre-study blood test assessments, and study subjects needed to stop taking antiphlogistic treatment, acupuncture or physiotherapy during this period. Eligible study subjects were randomly assigned to the treatment group and wait-list control group in 1:1 ratio. An independent researcher used the random number function in Microsoft Excel 2019 to generate the randomization sequence and preparing the sequentially numbered opaque sealed envelopes containing the random number and corresponding intervention assignment. After eligibility assessment, a researcher opened the envelopes and assigned the interventions correspondingly.

During the study period, study subjects were informed of their assigned group. However, Tui-na practitioners and statisticians were blinded to randomly allocated intervention but followed standard operation procedure (SOP).

### Interventions

Treatment group received the standardized Tui-na and DHJSD, while control group received Tui-na alone during the study period. Study subjects in the treatment group underwent 8 sessions of 20 min Tui-na with the same manipulation by well-trained independent, blinded Chinese medicine practitioner investigators with over 5 years of clinical experience, and took DHJSD orally twice daily concomitantly over 4 weeks. The control group only received standardized Tui-na in the same manner of the treatment group but was given the same regimen of DHJSD after the completion of week 8 follow-up assessments at study subjects’ discretion as a compensation. The Chinese medicine practitioners had ever received standard training on the Tui-na manipulation applied in this study.

The procedures of Tui-na manipulation were taken following a standard procedure [16]. In the beginning, the patient took a supine position. The trained Chinese medicine practitioner (Tui-na practitioner) applied rolling manipulation from the quadriceps femoris to the knee cap, and pressing, kneading both sides of knee cap for 5 min. Pressing and kneading Xuehai (SP10), Liangqiu (ST34), Heding (Ex-LE2), Dubi (ST35), Neixiyan (Ex-LE4) and Yanglingquan (GB34), 1 min for each. Later on, the patients took a prone position. The practitioner applied rolling manipulation from the bottom of the thigh to the top of the calf for 5 min. Weizhong (BL40) and Chengshan (BL57) were pressed, 1 min for each. Next, the patient took a supine position, with the affected knee and hip flexed. The Tui-na practitioner supported the upper part of the knee to fix it with one hand and held the heel with the other hand to rotate the knee joint clockwise and counterclockwise for 5 times. Then, the practitioner held the ankle and lifted the treated extremity. The lower extremity should be raised to form a 30° angle between the extremity and the bed. While pulling with slight force, shook constantly up and down in a small amplitude for 10 s. Finally, the practitioner rubbed the sides of the knee joint, the edge of knee cap and clearance for 15 s. Names and details of acupoints used in Tui-na manipulations are listed in Additional file 1: Table S1. The whole procedure of Tui-na manipulation cost about 20 min, and was conducted twice 1 week. Totally 8 sessions of Tui-na manipulation was conducted during treatment period of 4 weeks.

DHJSD was composed of Du-huo (Angelicae Pubescentis Radix) 10 g, Sang-ji-sheng (Taxilli Herba) 15 g, Du-zhong (Eucommiae Cortex) 10 g, Niu-xi (Achyranthis Bidentatae Radix) 12 g, Xi-xin (Asari Radix et Rhizoma) 1 g, Qin-jiao (Gentianae Macrophyllae Radix) 10 g, Fu-ling (Poria) 15 g, Fang-feng (Saposhnikoviae Radix) 10 g, Chuan-xiong (Chuanxiong Rhizoma) 6 g, Dang-shen (Codonopsis Radix) 15 g, Gan-cao (Glycyrrhizae Radix et Rhizoma) 6 g, Bai-shao (Paeoniae Radix Alba) 12 g, Di-huang (Rehmanniae Radix) 15 g, Ji-xue-teng (Spatholobi Caulis) 12 g and Cu-yan-hu-suo (Corydalis Rhizoma) 3 g. The concentrated Chinese medicine granules were used with corresponding dosage. For those patients with excess dampness pattern presenting heavy body, white and greasy tongue coating, additional herbs including Fang-ji (Stephaniae Tetrandrae Radix) 10 g, Yi-yi-ren (Coicis Semen) 15 g, and Cang-zhu (Atractylodis Rhizoma) 9 g were added. For those with blood stasis pattern presenting dark red or purple tongue, additional herbs including Tao-ren (Persicae Semen) 10 g and Hong-hua (Carthami Flos) 6 g were added. The pharmaceutical effects of the herbs in DHJSD were shown in Additional file 1: Table S2.

### Follow-up visits

Both groups are assessed at week 2, week 4 and were further followed up at week 8 (post 4 weeks intervention) with any symptoms of the knee and adverse events being assessed at each visit.

For safety concern, we recorded medical history and adverse events during the interventions and follow-up period, and administered appropriate treatment or referral if needed. Interventions were suspended immediately should a treatment-related serious adverse events occurred. Further assessments were needed to decide whether the trial should be suspended. There had a designated hotline for adverse event reporting. Study subjects could call the hotline during office hours but were advised to attend Emergency Department at the nearest hospital during non-office hours if severe adverse event was found. At the end of the intervention, the study subjects received post-study blood tests, included complete blood picture tests, liver and renal function tests for safety monitoring.

In order to encourage study subjects’ continued compliance, they were contacted by telephone 1 to 2 days prior to each visit. For the treatment group who had study medications, leftover packages were counted to monitor each study subject’s compliance. The consumption of topical drugs and non-steroidal anti-inflammatory drugs (NSAIDs) rescue medication were also recorded at each visit.

## Outcome measurements

### Primary outcome measure

The primary outcome measure was rated using the WOMAC in Chinese version [17] at 4 weeks after randomization. The WOMAC is a validated tool for self-administration. It is a 3-dimensional questionnaire with subscales assessing pain, stiffness and physical functional disability in study subjects with KOA using a series of 24 questions. It is a five-point scale from 0 to 4 (0 = none, 1 = mild, 2 = moderate, 3 = severe, 4 = extreme).

### Secondary outcome measures

Secondary outcome measured WOMAC total score in Chinese version at 2 weeks and 8 weeks after randomization, and also the pain, stiffness and physical functional disability subscales.

The EQ-5D-5L is used to assess health-related quality of life and particularly utility values [18, 19]. It shows high responsiveness in study subjects and it can also show important changes clinically [20]. The EQ-5D-5L essentially consists of two parts: the EQ-5D descriptive system and the EQ visual analogue scale (EQ VAS). The descriptive system comprises five dimensions: mobility, self-care, usual activities, pain/discomfort and anxiety/depression. Each dimension has five levels: no problems, slight problems, moderate problems, severe problems and extreme problems. The VAS can be used as a quantitative measure of health outcome that reflects the study subjects’ own judgment. It recorded the study subjects’ self-rated health on a vertical visual analogue scale. Outcomes were documented at 2 weeks, 4 weeks after randomization and follow-up (8 weeks).

## Ethics consideration

The study was approved by the Joint Chinese University of Hong Kong-New Territories East Cluster Clinical Research Ethics Review Committee (CREC Ref. No. 2019.538). And the study was registered at the ClinicalTrials.gov (website: https://clinicaltrials.gov/ct2/show/NCT04492670, registry number: NCT04492670).

## Study monitoring

A dedicated team from the Hospital Authority Chinese Medicine Department (HACMD) that was completely uninvolved in the running of the study and had no competing interests were responsible for study monitoring.

## Sample size calculation and statistical analysis

The sample size calculation was based on the primary outcome, the WOMAC. Based on a previous study, we assumed Tui-na combined with DHJSD could decrease WOMAC 17.44 with standard deviation 23.61 than the control group at week 4 [21]. The sample size was calculated according to the following formula.

$$n=\frac{2{\sigma }^{2}{\left({t}_{\alpha }+{t}_{\beta }\right)}^{2}}{{\left({\mu }_{1}-{\mu }_{2}\right)}^{2}}$$

The µ1 − µ2 was the difference in means 17.44. The σ was 23.61. We define α as 0.05 and β as 0.1, the t_α_ and t_β_ were 1.96 and 1.282 respectively. The sample size for each group was 29. Considering 20% drop-out, the total sample size was 70.

All effectiveness and safety analyses were conducted according to the intention-to-treat (ITT) principle. Missing values were imputed by the last-observation-carried forward method. The statistical analysis was performed using the Statistical Packages for the Social Sciences (SPSS) for Windows, version 27.0. Statistical significance was defined as a two-sided P value of < 0.05. Baseline characteristics were reported as mean ± SD.

Repeated measure of analysis of covariance (ANCOVA) was used to adjust the potential confounding variables on the outcomes. In this study, WOMAC Stiffness subscale score and WOMAC Physical Function subscale score (shopping) were potential confounding variables that were adjusted. The changes in scores from baseline to the end-point of treatment were tested using paired t-test.

## Results

### Study subjects and baseline characteristics

**Fig. 1:**
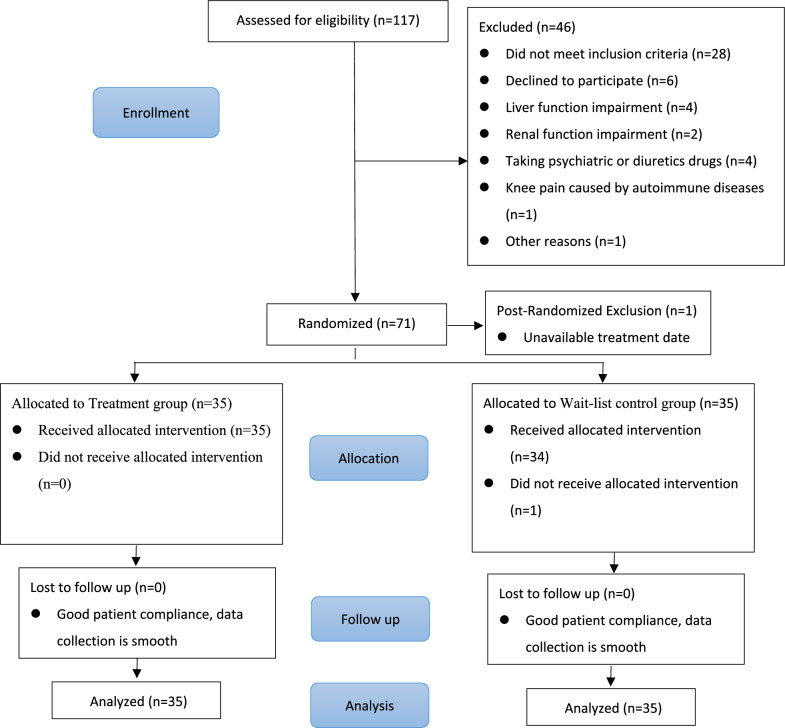
Flow chart of the study design

Between September 2020 to September 2021, 117 study subjects were screened according to the inclusion criteria, and 46 (39.3%) were excluded. 71 eligible study subjects were enrolled. 1 was post-randomization excluded due to unavailable treatment appointment. The remaining 70 study subjects were randomly divided into two groups. Among 70 randomly assigned study subjects, 1 from the control group was withdrawal due to working reason, all the rest completed the 4-week course of treatment and were assessed at week 8. A flow chart of this study is presented in Fig. 1. There was no significant difference between the two groups in age, gender, body mass index (BMI) and other demographic characteristics. Baseline characteristics of the study subjects are presented in Table 1. Before treatment, there was no significant difference in WOMAC total scores and the EQ-5D descriptive system as well as EQ VAS between two groups (P > 0.05).

**Table 1:** Baseline demographic characteristics

Characteristics	Treatment group (n = 35)	Wait-list control group (n = 35)
Age (years)	64.8 (7.5)	64.5 (5.9)
Sex, n (%)		
Male	9 (25.7)	6 (17.1)
Female	26 (74.3)	29 (82.9)
Weight (kg)	64.4 (11.3)	65.8 (12.1)
Height (cm)	157.0 (8.3)	156.0 (6.6)
BMI	26.1 (3.8)	27.1 (4.9)
Pain area, n (%)		
Left knee	20 (57.1)	19 (54.3)
Right knee	15 (42.9)	16 (45.7)
Pain duration (months)	99.1 (93.6)	101.1 (85.9)

Values are presented as mean (SD) except where noted

SD: standard deviation; BMI: body mass index (calculated as weight in kilograms divided by height in meters squared)

All 35 study subjects in the treatment group completed the whole Tui-na treatment (8 sessions). In the control group, 3 study subjects (8.57%) did not receive the whole Tui-na treatment. In the treatment group, 3 study subjects (8.57%) took less than 80% of the prescribed dosage over the study period.

All 35 study subjects in the treatment group were prescribed DHJSD. Eight of them (22.86%) manifested excessive dampness patterns at the baseline and were prescribed DHJSD with Fang-ji (Stephaniae Tetrandrae Radix), Yi-yi-ren (Coicis Semen) and Cang-zhu (Atractylodis Rhizoma), one of them did not manifest this pattern at week 2 and the remaining medication had changed to the basic study medication. Ten of them (28.57%) manifested blood stasis patterns at baseline and were prescribed DHJSD with Tao-ren (Persicae Semen) and Hong-hua (Carthami Flos), one of them did not manifest this pattern at week 2 and the remaining medication had changed to the basic study medication. One study subject (2.86%) manifested both excessive dampness and blood stasis pattern and was prescribed DHJSD with the five additional herbs.

### Primary and secondary outcomes

**Fig. 2:**
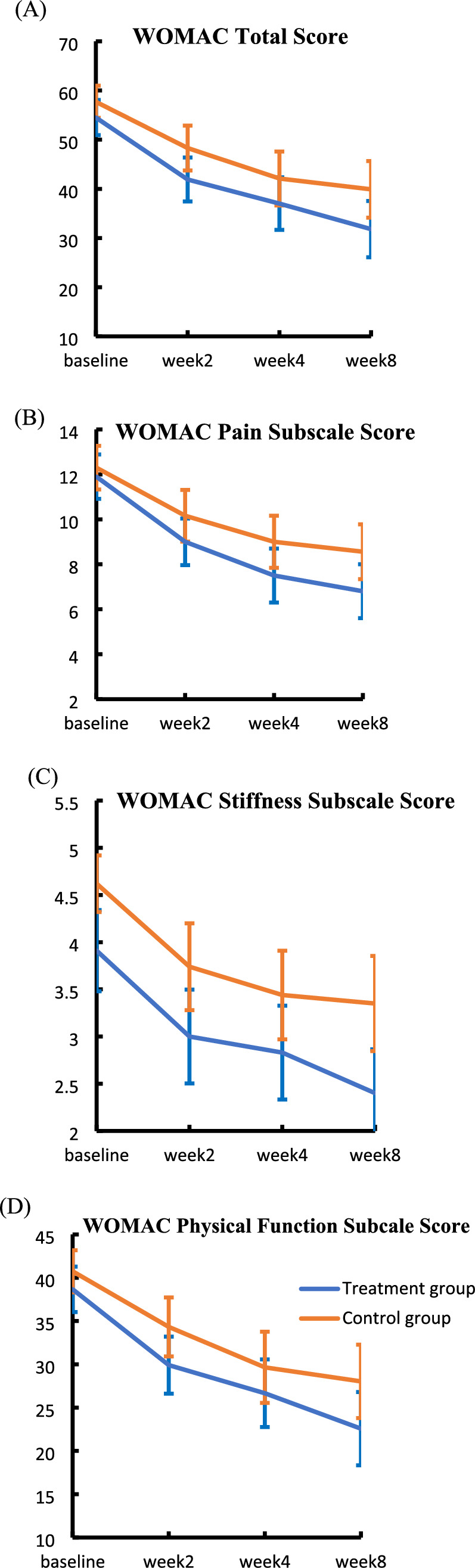
The WOMAC total score and subscale scores during the study period. **A** WOMAC total score; **B** Pain subscale score; **C** Stiffness subscale score; **D** Physical function subscale score

The WOMAC total scores in the treatment group and the control group were similar at 4 weeks with the mean of 37.0 compared with 42.1 (P = 0.2) (Table 2). However, the analysis of WOMAC total scores and all the subscales scores in both groups showed statistically significant improvement with time, when comparing baseline versus week 4 and baseline versus week 8 (P < 0.001 and P < 0.001 respectively) (Fig. 2). On comparison between two groups, the treatment group showed a trend of improvement than the wait-list control group.

**Table 2:** WOMAC and EQ-5D-5L score over time between groups, mean (SD)

	Treatment/control group (baseline)	Difference (95% CI, P value) (baseline)	Treatment/control group (week 2)	Difference (95% CI, P value*) (week 2)	Treatment/control group (week 4)	Difference (95% CI, P value*) (week 4)	Treatment/control group (week 8)	Difference (95% CI, P value*) (week 8)
WOMAC Total score	54.5 (10.8)/57.7 (9.7)	− 3.2 (− 8.1 to 1.8; 0.20)	41.9 (13.5) 48.3 (13.6)	− 6.4 (− 12.9 to 0.12; 0.05)	37.0 (16.2)/42.1 (16.3)	− 5.1 (− 12.9 to 2.7; 0.20)	31.8 (17.3)/39.9 (17.1)	− 8.2 (− 16.5 to 0.1; 0.05)
Pain subscale	11.9 (2.9)/12.3 (2.9)	− 0.41 (− 1.8 to 1.0; 0.57)	9.0 (3.1) 10.3 (3.4)	− 1.2 (− 2.8 to 0.34; 0.12)	7.5 (3.6)/9.0 (3.5)	− 1.5 (− 3.2 to 0.24; 0.09)	6.8 (3.6)/8.6 (3.6)	− 1.8 (− 3.5 to − 0.02; 0.048)*
Stiffness subscale	3.9 (1.3)/4.6 (0.9)	− 0.7 (− 1.3 to − 0.2; 0.013)*	3.0 (1.5) 3.7 (1.4)	− 0.7 (− 1.4 to − 0.05; 0.035)*	2.8 (1.5)/3.4 (1.4)	− 0.6 (− 1.3 to 0.08; 0.082)	2.4 (1.4)/3.4 (1.5)	− 1.0 (− 1.6 to − 0.3; 0.008)*
Physical function subscale	38.7 (7.9)/40.7 (7.1)	− 2.1 (− 5.7 to 1.6; 0.26)	29.9 (9.9) 34.3 (10.1)	− 4.4 (− 9.2 to 0.41; 0.07)	26.7 (11.8)/29.7 (12.2)	− 3.0 (− 8.8 to 2.8; 0.31)	22.6 (12.8)/28.0 (12.6)	− 5.5 (− 11.6 to 0.65; 0.08)
EQ-5D Index value	0.30 (0.29)/0.19 (0.27)	0.10 (− 0.03 to 0.24; 0.12)	0.47 (0.26) 0.30 (0.33)	0.17 (0.02 to 0.31; 0.022)*	0.51 (0.26)/0.38 (0.34)	0.13 (− 0.01 to 0.28; 0.07)	0.57 (0.30)/0.45 (0.29)	0.11 (− 0.03 to 0.25; 0.13)
EQ VAS	54.7 (16.6)/51.8 (19.1)	3.1 (− 5.5 to 11.7; 0.48)	62.6 (19.3) 59.1 (16.2)	3.5 (− 5.12 to 12.0; 0.42)	65.9 (14.0)/62.1 (18.6)	3.8 (− 4.1 to 11.7; 0.34)	65.7 (19.2)/62.9 (16.5)	2.9 (− 5.8 to 11.5; 0.51)

*Independent T-test

No significant difference was found between two groups on WOMAC subscales at the end of treatment (week 4). However, there was a statistical difference in mean WOMAC Pain subscale score at week 8 follow up between the two groups (P = 0.048). The mean difference (MD) was 1.76 with 95% confidence interval (CI) of 0.02 to 3.5. The treatment group demonstrated better improvement in pain relief than control group (Table 2). Besides, there were statistical differences of mean WOMAC Stiffness subscale scores between the two groups at week 2 (MD 0.74, 95% CI 0.05 to 1.42, P = 0.035) and week 8 (MD 0.95, 95% CI 0.26 to 1.65, P = 0.008) (Table 2).

For EQ-5D-5L, the analysis of EQ-5D index values and EQ VAS scores showed no difference between two groups at week 4 and week 8. There were statistical differences of mean EQ-5D index values for the two groups at week 2 (MD 0.17, 95% CI 0.02 to 0.31, P = 0.022). When comparing baseline versus week 4 and baseline versus week 8, EQ-5D index values and EQ VAS scores also showed statistically significant improvement with time from baseline (P < 0.001).

### Additional drugs

Both groups showed decreasing consumption of topical drugs and non-steroidal anti-inflammatory drugs (NSAIDs) during treatment. Twenty-one subjects (60.0%) from each group used additional drugs to relieve knee pain at the baseline. After the 8-week trial, only 4 (11.4%) and 5 (14.3%) study subjects consumed additional drugs in the treatment group and the wait-list control group respectively. There was significant difference in time for both groups (P < 0.001), but no significant difference between groups (P = 0.73).

### Safety outcomes

#### Adverse events

During the 8-week trial, there were no report on serious adverse events. 2 study subjects (5.7%) from the treatment group and 6 (17.1%) from the wait-list control group reported mild local pain in the lower limbs after receiving Tui-na manipulation. There was no significant difference in the occurrence of adverse events between two groups (P = 0.12). Besides, in the treatment group, seven (20.0%) adverse drug reactions were potentially related to the medication, involving change in stool frequency and abdominal distention. All these adverse events were mild and subsided in a short of time without medical care.

#### Laboratory investigations

After 4-week intervention, there were few abnormal findings in the liver function test. 2 study subjects (5.7%) from the treatment group and 3 (8.6%) from the control group showed alanine aminotransferase (ALT), aspartate aminotransferase (AST) or total bilirubin slightly increased in the blood tests. There was no significant difference in the occurrence of abnormal blood test results between two groups (P = 0.62). These study subjects showed no symptoms or signs related to the abnormal liver function and all returned to normal after having re-check blood tests 2 weeks later.

For other examinations, there were no significant changes in the vital signs, blood tests including complete blood picture tests and renal function tests after the intervention in both groups.

## Discussion

The present study demonstrated that there was improvement in pain relief, stiffness knee function, as well as QOL in both groups during the 4-weeks intervention. And the improvement kept on going at week 8 followed up. These results suggest that Tui-na or the combined treatment have positive effects in terms of WOMAC scores, as well as the quality of life of KOA patients.

The treatment group showed more improvement in WOMAC Pain subscale score at week 8, the results demonstrated Tui-na was effective for immediate relief of pain, while oral Chinese medicine as an add-on effect was effective in long-term pain relief. Besides, the treatment group showed significant improvements in WOMAC Stiffness subscale score at week 2 and week 8.

Tui-na, Chinese massage, is an important part of Tradition Chinese Medicine with more than 2000 years history [22]. It is a treatment based on meridian system. Practitioners use hands and arms to manipulate soft tissue, which stimulate the acupoints and mobilize joints by different techniques of Tui-na such as rolling, kneading and pulling to dredge the meridian system and promote the Qi and Blood circulation [23]. This method was used to treat different disorders, such as muscle weaknesses, and joint pains. Tui-na was included as a component of physiotherapy intervention for KOA in the 2018 Expert Consensus on Step Treatment of Knee Osteoarthritis statement in China [24]. It is one of the most popular therapies for KOA because it is a non-invasive treatment and is widely accepted by patients.

KOA is a degenerative disease and the most common cause of disability in the elderly [25]. The major symptoms of KOA include knee pain, muscle weakness and loss of joint function. Compare to current pharmacological treatment, Tui-na is more beneficial to the patients and health care system because of its simple operation, fewer side-effect and lower financial cost [26, 27].

Many studies had demonstrated that Tui-na is beneficial to KOA patients [28–30]. The results in this study are comparable to previous clinical trials. In this study, the quadriceps femoris and knee cap are manipulated. The function of quadriceps muscle is to maintain the stability of knee [31]. The weakness of quadriceps muscle is a common clinical sign of patients with KOA. The reason why manipulating the muscle is that massaging the quadriceps femoris not only can relieve pain, but also can improve the muscle strength and knee function in KOA patients [32–36]. Increased quadriceps strength also reduces the risk of symptomatic knee osteoarthritis [37]. The underlying mechanisms include: increased lymph and blood flow of the joint, increased clearance of blood lactate and absorption of inflammatory substances, promote the metabolism and nutrients supplication of articular cartilage [38–42].

In traditional Chinese medicine theory, there is a theory called muscle-region theory. A large proportion of muscles around the knee belong to the region of Foot Yang-ming. Patients with KOA showed a major damaged in the muscle region of Foot Yang-ming [43]. Hence, by rolling manipulated around this region, pressing and kneading the acupoints around the knee in this study, these can dredge the channels and promote the circulation of Qi and blood around the knee and disperses stasis. As a result, the channels are cleared and warmed [44, 45]. These manipulations were used to reduce swelling, break up adhesions and alleviates pain [22]. Besides, the knee joints were mobilized by rotating it clockwise and anti-clockwise respectively. The joints were then undergone a slight pulling and shaking. These manipulations were used to lubricate the joints, restoring the joints’ full range of movement, and help muscle relaxation by stimulating inhibitory reflex and Golgi tendon organ [46, 47].

In this study, herbal medication, DHJSD, was found to have add-on effects on reducing pain and stiffness after 4 weeks following the end of treatment and improving quality of life after 2 weeks of treatment. Our findings are similar to the relevant studies [48, 49]. Network pharmacology approaches demonstrated that DHJSD could reduce the immune-inflammatory reaction, inhibit apoptosis of chondrocytes, strengthen proliferation and repair of chondrocytes and reduce the inflammatory response in a multi-component–multi-target–multi-pathway way to play a role in the treatment of KOA [50–52].

Pattern differentiation is one of the most important treatment principles in the Chinese Medicine. Chinese medicine practitioners analyze patients’ signs and symptoms data through observation, listening, questioning and palpation, all information obtained will be summarized and differentiated to different patterns. Chinese herbal medicines are prescribed according to which patterns patients are.

According to traditional Chinese medicine theory, the most common pattern for KOA is kidney-yang deficiency with excessive dampness and blood stasis [10]. For patients related to kidney-yang deficiency, they may present knee pain with aching pain in lumbar vertebrae, deafness, hair loss, teeth loosen and urinary incontinence. For patients related to excessive dampness, they may present knee pain with heavy pain, heavy feeling in the body, pain worse in the rainy days and slippery tongue coating. For patients related to blood stasis, they may present knee pain with fixed stabling pain, purple lips and purple tongues [53].

DHJSD is used for patients with KOA diagnosed as kidney-yang deficiency pattern [10]. In our study, DHJSD was the basic formula for the treatment group. DHJSD aims to stop pain by dispelling wind-dampness, nourish the liver and kidney and tonify Qi and blood [54]. According to the Chinese herbology theory [55], Du-huo (Angelicae Pubescentis Radix), Qin-jiao (Gentianae Macrophyllae Radix) and Fang-feng (Saposhnikoviae Radix), each has the key function to dispel wind-dampness and release wind-cold. Xi-xin (Asari Radix et Rhizoma) is a warming herb with a remarkable pain-relieving power by disperse cold and wind. Chuan-xiong (Chuanxiong Rhizoma) and Cu-yan-hu-suo (Corydalis Rhizoma) both belong to blood-invigorating herbs which activate Qi and blood circulation and relieve pain. Research has found that alkaloids in Cu-yan-hu-suo (Corydalis Rhizoma) extracts are responsible for its anti-inflammatory effect [56]. Another study showed that Chuan-xiong (Chuanxiong Rhizoma) and Xi-xin (Asari Radix et Rhizoma) compounds demonstrate a synergistic analgesic effect [57]. Ji-xue-teng (Spatholobi Caulis) and Niu-xi (Achyranthis Bidentatae Radix) both activate blood circulation along with tonifying power. Ji-xue-teng (Spatholobi Caulis) would also tonifies blood while Niu-xi (Achyranthis Bidentatae Radix) nourishes liver and kidney and strengthens bones and tendons. Du-zhong (Eucommiae Cortex) and Sang-ji-sheng (Taxilli Herba) are often used in pair in many prescriptions because of their characteristics to tonify liver and kidney and strengthen bones and sinews. Dang-sheng (Codonopsis Radix), Gan-cao (Glycyrrhizae Radix et Rhizoma) and Bai-shao (Paeoniae Radix Alba) are tonic herbs to elevate Qi and blood level of the body.

Fang-ji (Stephaniae Tetrandrae Radix), Yi-yi-ren (Coicis Semen) and Cang-zhu (Atractylodis Rhizoma) were added if patients with excessive dampness patterns. Fang-ji (Stephaniae Tetrandrae Radix) can delay the progression of KOA by regulating lymphatic drain function [58], while Yi-yi-ren (Coicis Semen) and Cang-zhu (Atractylodis Rhizoma) can remove damp, relax muscles and tendon [59]. On the other hand, Tao-ren (Persicae Semen) and Hong-hua (Carthami Flos) were added if patients with blood stasis patterns. The function of Tao-ren (Persicae Semen) and Hong-hua (Carthami Flos) is to improve microcirculation [60].

In this study, the compliance rate was high, possibly because most of the study subjects were elderly and retired, with more time for treatment than those still working. Besides, they all lived close to the clinics, and many of them said that they found the interventions and the setting comfortable.

We found that DHJSD complementary to Tui-na for KOA were generally safe and well tolerated. The adverse events were mild, transient and disappear spontaneously without the need for discontinuing interventions. They are safe, relatively inexpensive, and effective way to relieve knee osteoarthritis.

There were several limitations to our study. Firstly, neither study subjects nor assessors were blinded to group allocation, possibly resulting in overestimation of the effects of the combined intervention. A third party to be assessor will be tried in the future studies. Secondly, there may be a selection bias as study subjects were enlisted through outpatient clinic-based recruitment and advertisements with posters and leaflets, they may be interested in caring for themselves and were more willing to participate. Thirdly, most study subjects were women as they are more active in community activities, this may have increased the gender bias. Fourthly, the treatment period of only 4 weeks was relatively short. Lastly, self-rating scales were used as outcome measurements; some study subjects may have recall bias when interpreting results.

Further studies are required to carry out in the future. Firstly, this study was conducted on a limited sample. It is desirable to conduct a similar study on larger samples. The information on some concomitant diseases such as diabetes and malnutrition should be collected. Secondly, increasing the duration of treatments and follow-up can help examine the clinical effect of DHJSD more reliably. Thirdly, it is valuable to use imaging examination, such as X-ray, Computed Tomography scan (CT) or magnetic resonance imaging (MRI), which can help to make diagnosis and also be one of the outcome measurements. Lastly, as Tui-na may also improve knee muscle function [36]. It is necessary to introduce objective outcomes to assess, such as knee muscle strength and balance using the Five-Times-Sit-to-Stand Test (FTSST) and the Timed Up and Go Test (TUG).

## Conclusion

The research findings have shown that the herbal medication of DHJSD may have add-on effect in pain relief, improving stiffness as well as QOL in patients with KOA and this combined treatment was generally safe and well tolerated. The findings highlight the potential of Tui-na and herbal medication as complementary treatment regimens for KOA. Large-scale clinical trials and double-blinded studies are needed to substantiate clinical evidence on the benefits of KOA and to identify the mechanisms underlying its efficacy. Availability of data and materials Data analyzed in this study can be obtained from the corresponding author upon request.

## Supplementary Information

**Additional file 1: Table S1.** Acupoints used in Tui-na manipulation. **Table S2.** The composition and the pharmacological effects of DHJSD.

## Data Availability

Data analyzed in this study can be obtained from the corresponding author upon request.

## Abbreviations

BMI: Body mass index
CREC: Clinical Research Ethics Review Committee
DHJSD: Du-Huo-Ji-Sheng Decoction
EQVAS: EQ visual analogue scale
EQ-5D-5L: 5-Level EQ-5D version
HACMD: Hospital Authority Chinese Medicine Department
ITT: Intention-to-treat
KOA: Knee osteoarthritis
QOL: Quality of life
SD: Standard deviation
SOP: Standard operation procedure
SPSS: Statistical Packages for the Social Sciences
TKR: Total knee replacement
WOMAC: Western Ontario and McMaster University Osteoarthritis Index
