# Supplementary material for: Therapeutic effect of Duhuo Jisheng Decoction add-on Tui-na manipulation on osteoarthritis of knee: a randomized controlled trial
Source: Chin Med. 2023 Jul 10;18:82. doi: 10.1186/s13020-023-00737-5 (PMC10331987; doi:10.1186/s13020-023-00737-5)
Supplement: Supplementary file 1 — Additional file 1: Table S1. Acupoints used in Tui-na manipulation. Table S2. The composition and the pharmacological effects of DHJSD. [file 13020_2023_737_MOESM1_ESM.docx]

**Table S1.** Acupoints used in Tui-na manipulation

| **Name of acupoints** | **Areas** | **Special qualification** | **Indications** |
| --- | --- | --- | --- |
| Xuehai (SP10) | When the knee is flexed, 2 cun above the medical superior border of the patella on the bulge of the medial portion of m. quadriceps femoris | - | Irregular menstruation, heavy uterine bleeding, amenorrhea; urticaria, eczema, erysipelas; abdominal distension, diarrhea, jaundice; abdominal pain, difficulty in micturition, edema; pain and swelling in the knees |
| Liangqiu (ST34) | On the line connecting the anterior superior iliac spine and the lower lateral border of the patella, 2cun above the upper lateral border of the patella | Xi- Cleft Point | Stomach pain; knee pain, atrophy and paralysis of the legs; mastitis |
| Heding (Ex-LE2) | Above the knee, in the depression of the midpoint of the superior patellar border | - | Knee pain, weakness of the leg and foot, and palsy |
| Dubi (ST35) | When the knee is flexed, at the lower border of the patella, in the depression lateral to the patella ligament | - | Swelling and pain in the knees, difficulty in flexing and extending the knees and beriberi |
| Neixiyan (Ex-LE4) | In the depression medial to the patellar ligament when the knee is flexed | - | Pain in the knee, atrophy or paralysis of the lower limbs |
| Yanglingquan (GB34) | In the depression anterior and inferior to the small head of the fibula | He-Sea Point; Lower He-Sea Point of the Gallbladder; One of the Eight Influential Points | Jaundice, bitter taste in mouth, hiccups, vomiting; pain in the hypochondriac region; swelling and pain of the knee, atrophy or paralysis in the lower limbs |
| Weizhong (BL40) | On the midpoint of the transverse crease of the popliteal fossa, between the tendons of the biceps femoris and semitendinous | He-Sea Point; Lower He-Sea Point of the Bladder | Lumbar pain, spasm of the popliteal tendons, weakness or paralysis in the lower limbs; difficulty in urination, enuresis; erysipelas, urticaria,, furuncles |
| Chengshan (BL57) | In the centre of the posterior aspect of the lower leg, between Weizhong (BL40) and Kunlun (BL60), in the triangle depression formed below the bellies of the gastrocnemius muscle when the foot is stretched | - | Pain and spasm in the lumbar region and legs; haemorrhoids, constipation |

*According to "Acupuncture and Moxibustion" edited by Shi XM, (China Press of Traditional Chinese Medicine, 2002)

**Table S2.** The composition and the pharmacological effects of DHJSD

| *Angelicae Pubescentis Radix* | TCM: Dispels wind, eliminates dampness, frees cold, stops pain. | |
| --- | --- | --- |
|  | Pharmaceutical study: Apply to wind-cold and damp impediment, pain of waist and knees, subside wind of Shao Yin and headache, headache and toothache. | |
| *Taxilli Herba* | TCM: Dispels wind-damp, supplements liver and kidney, strengthens sinew and bone, secures fetus. | |
|  | Pharmaceutical study: Apply to cold pain in the waist and knees, weakness of bones and muscles due to flaccidity, hemiplegia, rheumatic arthralgia, lightheadedness and dizziness, restless fetal movement, metrorrhagia and metrostaxia. | |
| *Eucommiae Cortex* | TCM: Supplements the live and kidney, strengthens sinew and bone, calms the fetus. | |
|  | Pharmaceutical study: Apply to aching pain in the loins and knees, weakness of legs and knees, drenching and unsmooth urination, vaginal dampness and itch, fetal leakage and abortion, restless fetal movement, hypertension. | |
| *Achyranthis Bidentatae Radix* | TCM: Supplements liver and kidney, strengthens sinew and bone, invigorates blood, frees channels, conducts the blood to move downward, promotes urination. | |
|  | Pharmaceutical study: Apply to sour pain of waist and knees, tired and soft of lower limbs, blood stasis and amenorrhea, dysmenorrhea, abdominal pain after giving birth, aggregation-accumulation, retention of placenta after giving birth, heat strangury, blood strangury, injury of trauma, abscess swelling and sore, throat swelling and pain. | |
| *Asari Radix et Rhizoma* | TCM: Resolvers exterior, disperses cold, dispels wind, stops pain, warms lung, transforms fluids, frees nose. | |
|  | Pharmaceutical study: Apply to exterior disease induced by pathogenic wind-cold, headache, toothache, wind-damp impediment, dyspnea with cough due to phlegm and retained fluids, nasal obstruction, rhinorrhea with turbid discharge, mouth sore. | |
| *Gentianae Macrophyllae Radix* | TCM: Dispels wind-damp, comforts sinews, clears deficiency heat. | |
|  | Pharmaceutical study: Apply to wind-damp impediment, contracture of bones and muscles, sour pain of bone and joint, afternoon tidal fever, infantile malnutrition with fever. | |
| *Poria* | TCM: Promotes urination, leaches out dampness, fortifies the spleen and stomach, calms spirit. | |
|  | Pharmaceutical study: Apply to dysuria, edema, phlegm and retained fluid and cough, vomiting, less intake due to deficiency of spleen, diarrhea, and fright and nervous, insomnia and amnesia, spermatorrhea and gonorrhea. | |
| *Saposhnikoviae Radix* | TCM: Resolves the exterior, disperses wind, eliminates dampness, relieves tetany. | |
|  | Pharmaceutical study: Apply to exterior syndrome due to wind-cold, headache, dizziness, rigid neck, rheumatic arthralgia with wind-cold, joint pain and sour, convulsion of limbs, tetanus. | |
| *Chuanxiong Rhizoma* | TCM: Invigorates the blood, eliminates stasis, moves Qi, opens stagnation, dispels wind, relieves pain. | |
|  | Pharmaceutical study: Apply to chest impediment and heartache, chest pain, swollen pain of falls wound, irregular menses, amenorrhea, dysmenorrheal, abdominal mass and pain, headache, wind-damp impediment. | |
| *Codonopsis Radix* | TCM: Supplements the center, boosts Qi, fortifies the lung, boosts the spleen. | |
|  | Pharmaceutical study: Apply to weakness of spleen and lung, short breath and palpitations, anorexia and loose stool, dyspnea and cough due to deficiency of the lung, feverish dysphoria and diabetes. | |
| *Glycyrrhizae Radix et Rhizoma* | TCM: Supplements the spleen, boosts Qi, clears heat, resolves toxin, dispels phlegm, relieves cough, relaxes tension, relieves pain, harmonizes the nature of other medicinals. | |
|  | Pharmaceutical study: Apply to weakness of the spleen and stomach, weariness and debilitation, coughing with asthma, excessive phlegm, palpitation and shortness of breath, pain of gastric cavity, contraction and pain in four limbs, anthracia and sores, relieving drug toxicity, severity of toxicity. | |
| *Paeoniae Radix Alba* | TCM: Nourishes the blood, regulates menstruation, calms the liver, relieve pain, astringes yin, stops sweating. | |
|  | Pharmaceutical study: Apply to thoracic, abdominal and costal pains, abdominal pain due to dysentery, spontaneous perspiration and night sweat, fever with yin deficiency, irregular menstrual periods, metrorrhagia and metrostaxis, leukorrhea. | |
| *Rehmanniae Radix* | Sheng di haung | TCM: clears heat, cools blood, nourishes yin, and engenders fluids. |
|  |  | Pharmaceutical study: Apply to acute pyreticosis, fever and coma, macular eruption, hydrodipsia due to depletion of body fluids, hematemesis due to hemopyretic bleeding, non-traumatic hemorrhage, metrorrhagia and metrostaxis, hemafecia, mouth and tongue ulcers, swelling pain in the throat, coughing with over-strained fever, injury pains from falls, anthracia. |
| *Spatholobi Caulis* | TCM: Supplements blood, invigorates blood, frees network vessels. | |
|  | Pharmaceutical study: Apply to irregular menstrual periods, hematasthenic flaccidity, numbness and paralysis, rheumatic arthralgia. | |
| *Corydalis Rhizoma* | TCM: Invigorates blood, disperses stasis, moves Qi, stops pain. | |
|  | Pharmaceutical study: Apply to chest impediment and heart pain, flank and abdomen pain, headache, waist pain, hernis pain, sinew and bone pain, dysmenorrhea, amenorrhea, postpartum blood stasis and abdominal pain, injury from falls. | |
| *Stephaniae Tetrandrae Radix* | TCM: Promotes urination, reduces swelling, dispels wind, relieves pain. | |
|  | Pharmaceutical study: Apply to edema and beriberi, difficulty in micturition, wind-damp impediment, rheumatic arthralgia, hypertension. | |
| *Coicis Semen* | TCM: Fortifies the spleen, percolates dampness, eliminates impediment, relieves diarrhea. | |
|  | Pharmaceutical study: Apply to edema, beriberi, dysuria, damp arthralgia and contracture of tendons and vessels, diarrhea due to spleen deficiency. | |
| *Atractylodis Rhizoma* | TCM: Dries dampness, fortifies the spleen, dispels wind, disperses cold, and brightens the eyes. | |
|  | Pharmaceutical study: Chest congestion and abdominal distention, diarrhea and edema, beriberi and paralysis, wind and dampness impediment, wind-cold, caecitasnocturna. | |
| *Persicae Semen* | TCM: Invigorates blood, dispels stasis, moistens intestines, frees stools, relieves cough, calms panting. | |
|  | Pharmaceutical study: Apply to amenorrhea, dysmenorrhea, abdominal mass, injury from falls, and constipation due to dryness of intestines. | |
| *Carthami Flos* | TCM: Boosts blood, frees channels, disperses stasis, stops pains. | |
|  | Pharmaceutical study: Apply to menostasia, painful menstruation, retention of the lochia, abdominal mass, chest impediment and pain, abdominal stagnation and pain, chest and flank sting, traumatic injury, sore swelling pain. | |
| TCM: Traditional Chinese Medicine | | |

*According to Hong Kong Baptist University Chinese Medicinal Material Images Database, Medicinal Plant Images Database and Chinese Medicine Specimen Database.
